# Effect of habitual consumption of Ethiopian Arabica coffee on the risk of cardiovascular diseases among non-diabetic healthy adults

**DOI:** 10.1016/j.heliyon.2020.e04886

**Published:** 2020-09-15

**Authors:** Gizaw Mamo Gebeyehu, Dereje Getachew Feleke, Meseret Derbew Molla, Tesfahun Dessale Admasu

**Affiliations:** a Arsi University, College of Health Sciences, Department of Biomedical Science, Asella, Ethiopia; b Addis Ababa University, College of Medicine and Health Science, Department of Biochemistry, Addis Ababa, Ethiopia; c University of Gondar, College of Medicine and Health Sciences, Department of Biochemistry, Gondar, Ethiopia

**Keywords:** Food science, Food analysis, Cardiology, Coffee, Lipid, Cardiovascular diseases, Fatty acids, Lipoprotein

## Abstract

**Background:**

Globally, coffee is one of the most consumed beverages and recently, it has been a target of researchers to understand its effect on human health whether good or bad. Even though there is controversy on coffee consumption effect in cardiovascular diseases, several reports pointed out that coffee has a positive effect on the occurrence and progression of chronic non-communicable diseases including cardiovascular diseases. However, the impact of Ethiopian coffee Arabica consumption on cardiovascular diseases has not been well investigated thoroughly.

**Objective:**

The aim of the present study was to investigate the effect of habitual consumption of Ethiopian Arabica coffee on the risk of cardiovascular diseases among non-diabetic individuals in Addis Ababa.

**Materials and methods:**

A cross-sectional study was conducted in 70 healthy individuals in Addis Ababa. The participants were 35 coffee drinkers (16 males; 19 females) and 35 non-drinkers (15 males; 20 females). Coffee consumption and demographic data were obtained by using questionnaires. Anthropometric measurements were measured according to World Health Organization standards. Blood samples were collected by trained laboratory technicians through aseptic and sterile techniques for the analysis of biochemical parameters. Serum was separated via centrifugation and transported to Addis Ababa University, College of Health Sciences, Biochemistry laboratory with an ice pack for analysis or stored at -80 °C. Results were compared between coffee consumers and non -consumers using appropriate statistical parameter.

**Result:**

The main finding of this study was that consumption of Ethiopian origin Arabica coffee leads to a significant increase in serum free fatty acids (FFAs) and high density lipoprotein (HDL) as well as a significant decrease in triacylglycerides (TAGs) but has no significant effect in both total cholesterol (TC) and low density lipoprotein (LDL). The magnitude of the effect is similar in both sexes.

**Conclusion:**

The present study demonstrated that Ethiopian coffee Arabica consumption significantly affected most serum lipid levels and so it may be possible to say it has a protective effect against risks of cardiovascular diseases (CVDs). However, the correlations between coffee consumption habits and serum lipid levels require further investigation through experimental and epidemiological studies with larger sample size, including different age groups and nutritional habits.

## 1 Introduction

Ethiopia is believed to be the original birthplace of coffee. The coffee plant, Coffee arabica, originates in Ethiopia. Legend has it that an Ethiopian goatherder, Kaldi, discovered coffee after noticing that his goats became a little extra energized after eating certain berries found on his land. And so, coffee was born in a place called Kaffa, southern Ethiopia. Coffee beans from soils of Ethiopia are some of the most unique and alluring. Several reports indicate that dietary foods and beverages are the main determinant factors for the increment of chronic non-communicable diseases. Coffee as the most popular beverage in the world, including Ethiopia, is one of the dietary factors with controversial findings on its effect on health and disease. Coffee has different bioactive compounds that have long term effects on the risk of chronic non-communicable diseases including CVDs (O'Keefe et al., 2013). These compounds include caffeine, cafestol, kahweol phenolic acids, and diterpene alcohols that could have a positive and/or negative impact on health (Ludwig et al., 2014). The effects of coffee or its bio-active components on type 2 diabetes, cardiovascular disease, hypertension, neurological diseases, cancer, hormonal changes, gallstones, as well as renal stones have been studied through epidemiological, clinical, or experimental researches (Hu et al., 2001). As coffee consumption increases rapidly, understanding the effect of coffee consumption on the risk of CVDs has become an important target area for researchers.

A number of meta-analyses have reviewed the associations between coffee consumption and risk of CVDs (Cai et al., 2012; Wu et al., 2009; Mostofsky et al., 2012). Overall; many reports conclude that there is no association between coffee drinking and an increased risk of CVDs (Karabudak et al., 2015; Haffner et al., 1985; Richardson et al., 2009). In fact, several studies argued that a moderate intake of coffee has protection against the risk of CVDs (Orozco-Beltran et al., 2017). Additionally, one other report stated that long-term coffee consumption had modestly reduced risk of CVDs especially, stroke (Lopez-Garcia et al., 2009). In contrast, other report showed that there was a direct connection between drinking coffee and heart disease (Adebayo et al., 2007). However, the reports on the effect of coffee consumption on lipid profiles are contradictory (Karabudak et al., 2015; Cai et al., 2012). For example, metanalysis of twelve studies from western countries revealed a positive dose response relation between coffee consumption and level of TC, LDL and TAG (Cai et al., 2012). On the contrary, another population-based study in Turkey found that there is no association between coffee consumption and serum lipid profile (Karabudak et al., 2015). Furthermore, it has also been shown that unfiltered coffee increased TC and LDL cholesterol whereas filtered coffee has no significant effect (Jee et al., 2001). A lipidomic approach study reported that coffee consumption has no effect on lipid metabolites including free fatty acids and triglycerides but coffee drinking decreases lysophosphatidylcholine (Kuang et al., 2018).

These inconsistencies may originate from discrepancies in study design and/or brewing methods. In addition to this, the type of coffee is important factor. Ethiopian originated Arabica coffee is relatively rich in antioxidants and therefore it may have a protective effect against the risk of CVDs (Agudelo-Ochoa et al., 2016). However, to the best of our knowledge, the effect of Ethiopian Arabica coffee on the risk of CVDs on apparently healthy individuals is not well-studied. Therefore, the hypothesis of this study was that Ethiopian originated Arabica coffee consumption may prevent dysregulation of lipid profile (TAG, FFA, TC, HDL and LDL) and may have positive effect on insulin activity, and thereby provide protection against the risk of CVDs. Here, the effect of Ethiopian originated arabica coffee consumption on lipid profile (TAG, FFA, TC, HDL and LDL) was determined to indicate the risk of CVDs with their respective control groups.

## 2 Methods

### 2.1 Study population

The study population consisted of 70 subjects (31 males and 39 females) who were inhabitants of Addis Ababa. The sample size was calculated based on the following formula.

(1) N_1_ = N_2_ = [(1 + 1/r) (Z_•/2_ + Z•)^2^ (•^2^)]/•μ

where: N_1_-number of coffee drinkers, N_2_-number of non-coffee drinkers, r = N_1_/N_2_, Z_•/2_ = 1.96 at 5% type I error, Z_•_ = 0.84 at 80% po, N-Sample size = N_1_+N_2_ = 70, N_1_ = N_2_ = 35.

Study participants ages ranged between 21 and 49 years, with a mean age of 28.5 ± 7.5 years. Participants were required to meet the following criteria: no chronic diseases, including diabetes, coronary heart disease, cerebrovascular disease, hypothyroidism or other major diseases. Further, all participants were on a common Ethiopian dish with no extensive physical activity.

### 2.2 Study design and data collection

A cross-sectional study was conducted from October 2017 to June 2018 in Addis Ababa, Ethiopia. Data was collected through a face-to-face interview method. The dose of coffee consumption and demographic characteristics of the study participants were addressed with questionnaires.

The dose of coffee consumption was measured with Arabic cups of coffee per day. Coffee cup used by the study participants was estimated to be equal to 100ml. Participants were asked about the number of cups of coffee they drink per day and categorized as none, low-drinker (1–2 cups), moderate-drinker (3–4 cups) and high-drinker (≥5 cups). Unfiltered coffee has been reported to contain higher amounts of cafestol and kahweol and to increase TAG and LDL cholesterol levels (Rustan et al., 1997). Therefore, participants who consumed coffee prepared by filtered coffee brewing method only were included. Anthropometric measurements, including body weight (kg) and height (m), were measured according to the standards for each study participant, then body mass index (BMI) was calculated. BMI was calculated as the weight/height^2^ (kg/m^2^) and was classified according to the BMI cut-off points adopted by the World Health Organization. Blood pressure was assessed with a standard mercury sphygmomanometer after participants were allowed to sit for at least 5 min. Individuals with prehypertensive (SBP 130–139mmHg or DBP 80–89 mmHg), or hypertensive (SBP ≥140mmHg or DBP ≥90mmHg) were excluded from the study.

### 2.3 Blood sample collection and handling

Five milliliters (5ml) of blood was drawn from each study participant by venipuncture from the medial cubital vein, after overnight fasting. The participants were given to drink 75g of glucose dissolved in water and another blood sample was drawn after 2 h. All blood samples were taken by a trained laboratory technician using universal precautions. Blood samples were left to clot, and serum was separated within 2 h of collection by centrifugation for 10 min at 3000 rpm, then serum was transported to Addis Ababa University, Biochemistry laboratory for analysis. The aliquoted serum samples were stored frozen at -80 °C until analysis.

### 2.4 Methods of biochemical assays

Serum concentrations of FFA, TC, TAG, HDL, LDL and very low density lipoprotein (VLDL) were quantified (ml/dl) at Addis Ababa University, in Biochemistry laboratory. The level of these parameters in the serum was estimated by an enzymatic colorimetric method using a commercial kit supplied from Spinreact, Spain. All reagents used for the assay were prepared in the Biochemistry Laboratory, except ready-made kits for enzyme colorimetric assays. Colorimetric determinations for all parameters were conducted by microplate reader 2001 (Anthos Labtec instruments; Austria).

### 2.5 Statistical analysis

Prism 5.0 statistical package (Graph Pad Software, Inc, San Diego, CA, USA) and SPSS version 20 were used to analyze data from questionnaires and laboratory analyses. Descriptive data are presented with tables or graphs, and the data were expressed as frequencies/percentage, median, mean, standard error of the mean (SEM), interquartile range and p-value. The level of statistical significance was set at P-value <0.05.

### 2.6 Ethics approval and consent to participate

The study was approved by the Institutional Review Board of Biomedical Sciences, Arsi University. Written informed consent for each study participant was documented before data collection and drawing of blood samples. The right to withdraw from the study was kept at any time for the participants. Laboratory results were filled with codes and confidentiality was kept for all study participants. All methods were carried out in accordance with relevant guidelines and regulations.

## 3 Results

### 3.1 Characteristics of the study participants

Habitual coffee drinkers and non-coffee drinkers as control were included in this study. Habitual coffee consumers <65 years of age, residing in Addis Ababa, Ethiopia, with no family history of type 2 diabetes (T2D), were eligible for participation. Of the 75 participants recruited, 70 completed the trial. Five participants were excluded because of different reasons. Of the 70 participants completed the study, 55.7% (39) women, and 44.3% (31) men. About 35 of them were coffee drinkers whereas the other 35 were non-coffee drinkers. The majority of the study participants were within the age group of 20–25 years accounting for 38.6% followed by 26–30 years accounting for 27% and the least were in the age group greater than 35 years accounting for 14%. The highest proportions of coffee drinkers and non-drinkers were also observed within the age group of 20–25 years but there was no significant difference between the two groups, 40% and 37% for the coffee consumers and non-consumers respectively (Table 1).

**Table 1:** Age and gender distribution of study participants (N = 70), Addis Ababa, Ethiopia, 2018.

Age (year)	Sex		Participants∗		Total
	Male	Female	Coffee drinker	Non-coffee drinker	
20 to 25	19 (27.1%)	8 (11.5%)	14 (20%)	13 (18.6%)	27 (38.6%)
26 to 30	13 (18.6%)	6 (8.5%)	8 (11.4%)	11 (15.7%)	19 (27.1%)
31 to 35	6 (8.5%)	8 (11.4%)	7 (10.0%)	7 (10%)	14 (20.0%)
36 and above	1 (1.4%)	9 (12.9%)	6 (8.6%)	4 (5.7%)	10 (14.3%)
Total	39 (55.7%)	31 (44.3%)	35 (50%)	35 (50%)	70 (100%)

∗ To control confounding factors, all study participants (coffee drinkers and non-drinkers) were nonsmokers, didn't drink alcohol, had no family history of diabetes. In addition, participants who have common dietary habits and similar foods were included.

### 3.2 Grades of coffee consumption with respect to sex and BMI of the study participants

About 51.3% of the total non-coffee drinkers and 48.5% of the total coffee drinkers were females and the rest were males for both groups. Among the coffee drinkers, the highest proportion were in the category of low coffee drinkers while the least proportion were in heavy drinkers accounted for only 5.1% and 3.2% of the total females and males. The mean ± SD BMI of the coffee drinkers and non-drinker participants in the study was 20.6 ± 2.3 and 20.9 ± 2.4 respectively. It was found with a range of 17.0–27.0 in coffee drinkers and 17.0 to 25.0 in non-coffee drinkers. In addition, there was no statistically significant difference between the mean of BMI (P > 0.05) of different coffee drinker groups (Table 2).

**Table 2:** Coffee consumption grades with respect to sex and BMI of the study participants (N = 70), Addis Ababa, Ethiopia, 2018.

Coffee consumption∗	Total, N (%)	Sex, N (%)		BMI		P value
		Male	Female	Mean ± SD	Range	
Coffee drinker	35 (50%)	16 (22.9%)	19 (27%)	20.6 ± 2.3	17.0–27.0	P > 0.05
Low drinker	20 (28.6%)	8 (11.5%)	12 (17.1%)	20.5 ± 1.9	17.0–24.0	
Moderate drinker	12 (17.1%)	7 (10%)	5 (7.1%)	20.0 ± 2.4	17.0–24.0	
High drinker	3 (4.3%)	1 (1.4%)	2 (2.9%)	22.6 ± 3.5	20.0–25.0	
Non-drinker	35 (50%)	15 (21.4%)	20 (28.6%)	20.9 ± 2.4	17.0–25.0	

∗ The participants were classified based on their daily coffee consumption. Coffee consumption was measured with Arabic cup of coffee; Low drinkers were consuming 1–2 cups of coffee per day, Moderate drinkers were consuming 3–4 cups of coffee per day, High drinkers were consuming five and more cups of coffee per day and Non-drinkers were those participants who didn't consume or consuming less than one cup of coffee per day. P value of coffee drinker vs non-drinker and each subgroup vs non-drinkers.

### 3.3 Concentration of serum fasting and post-load insulin

There was no statistically significant difference between the median of fasting ((P > 0.05) and post loading serum insulin level (P > 0.05) of different coffee groups. Moderate-coffee drinkers showed slightly higher serum fasting insulin level, whereas heavy coffee drinkers appeared to have low post-loading insulin than other groups (Table 3).

**Table 3:** Concentration of serum fasting and post-load insulin for coffee consuming groups (N = 35), Addis Ababa, Ethiopia, 2018.

Variable	Low drinker (n = 20)	Moderate drinker (n = 12)	High drinker (n = 3)	Non-drinker (n = 35)
Fasting insulin, μU/l				
Median (1^st^ - 3^rd^ quartile)	15.2 (10.4–19.0)	13.9 (11.0–20.5)	15.0 (9.8–15.3)	12.7 (10.5–16.2)
Range	8.9–26.6	6.3–40.1	9.8–17.5	8.9–54.4
P value	ns	-	-	-
Post load insulin, μU/l				
Median (1^st^ - 3^rd^ quartile)	28.6 (22.6–36.4)	41.5 (27.1–55.9)	19.8 (12.6-?)	26.7 (19.0–45.0)
Range	14.8–65.3	20.4–104.4	12.6–50.2	12.5–102.5
P value	ns	-	-	-

### 3.4 Homeostasis model assessment for insulin resistance (HOMA-IR)

Homeostasis model assessment analysis for fasting insulin and glucose showed that there was no statistically significant difference between non-coffee drinkers and drinkers (P > 0.05).

**Figure 1:**
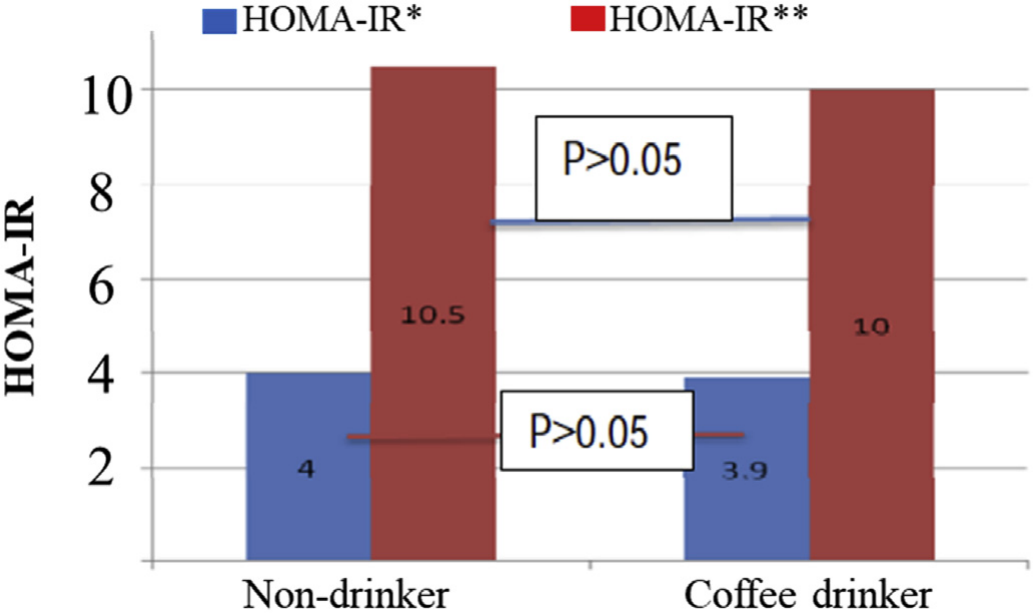
Homeostasis model assessments value for coffee drinker and nondrinker. HOMA∗ = Homeostasis model assessment for fasting insulin and glucose HOMA∗∗ = Homeostasis model assessment for post-loading insulin and glucose.

The same result was observed for HOMA-IR analysis for post-loading glucose and insulin (P > 0.05) (Figure 1).

### 3.5 Coffee consumption decreases serum TAG level

**Figure 2:**
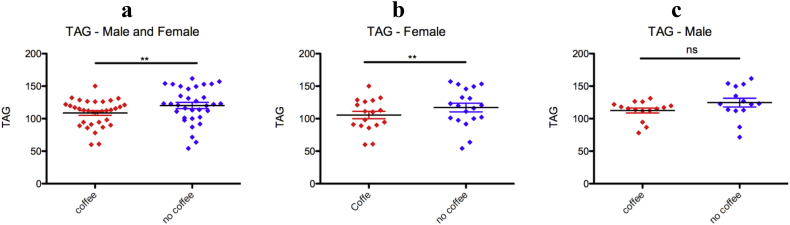
Coffee consumption decreases plasma TAG levels. (a) Coffee drinkers have lower plasma TAG levels compared to nondrinkers. (b) Coffee consumption decreases female's serum plasma TAG levels. (c) Coffee consumption has no effect on the male's serum TAG levels. (mean ± SD, unpaired T-test), ∗∗p < 0.01.

Habitual coffee drinkers had lower TAG levels compared to the nondrinkers (Figure 2a). Therefore, Ethiopian Arabica coffee consumption decreases serum TAG levels. To determine whether the effect of coffee on TAG is dependent on gender, data were subcategorized based on sex. Based on this category, the effect is statistically significant only in females but not in males (Figure 2b and c). Therefore, coffee consumption decreases the TAG level in females only. However, generally the TAG level of male coffee drinkers is also lower than the non-drinkers even though statistically insignificant. It would be interesting to investigate if sex hormones affect the effect of coffee on the lipid profile.

### 3.6 Coffee consumption increases plasma free fatty acid (FFA) level

**Figure 3:**
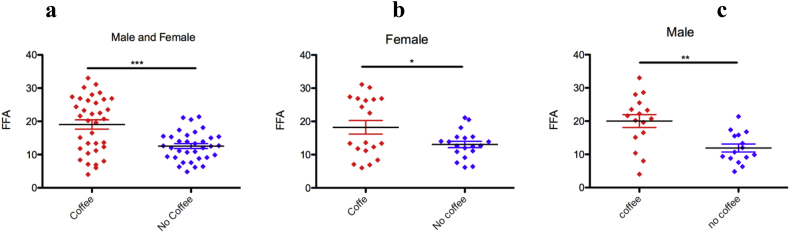
Coffee consumption increases plasma free fatty acid levels. (a) Coffee drinkers have higher plasma free fatty acid levels. (b) Coffee consumption increases female's serum plasma free fatty acid level. (c) Coffee consumption significantly increases serum plasma free fatty acid levels in males. (mean ± SD, unpaired T-test), ∗∗∗p < 0.001, ∗∗p < 0.01, ∗p < 0.05.

One of the possible mechanisms for a decrease in TAG, following coffee consumption, is it may be broken down to its components which leads to an increase in FFA. If this is the case plasma FFA level should increase following coffee consumption. To determine the effect of coffee consumption on FFA level serum plasma levels of free fatty acids were determined by enzymatic assay, as detailed in the method section, both in coffee drinkers and non-coffee drinkers. Interestingly, in agreement with our hypothesis coffee consumption increases the plasma level of free fatty acids (Figure 3a). When the data subcategorized based on gender, coffee consumption increased serum plasma free fatty acid levels of both males and females (Figure 3b and c). The effect size is more severe in males relative to females. Therefore, coffee consumption might stimulate TAG release and metabolism.

### 3.7 Coffee consumption increases HDL-cholesterol

**Figure 4:**
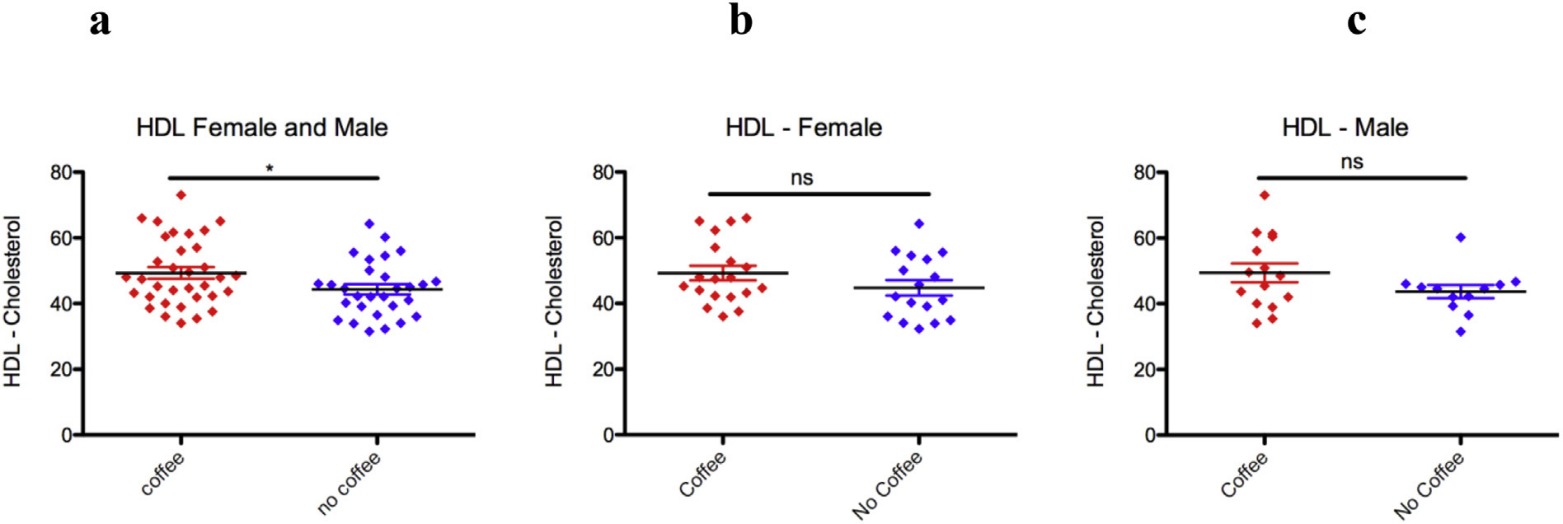
Coffee consumption increases HDL-cholesterol. (a) Coffee drinkers have a higher HDL-cholesterol level. (b) Coffee consumption has no effect on female and male (c) serum HDL-cholesterol levels. (mean ± SD, unpaired T-test), ∗p < 0.01.

To determine the effect of coffee consumption on the risk of CVD, cholesterol level was determined among coffee consumers and healthy controls. Habitual coffee consumers had higher HDL-cholesterol levels than the nondrinkers (Figure 4a). However, the statistics were significant only when males and female's data combined (Figure 4b and c). This could be due to our small sample size. Therefore, coffee consumption may have an advantageous effect on the risk of CVD. However, the association of coffee consumption and low risk of CVD remains to be determined in large sample size population-based studies.

### 3.8 Coffee consumption has no effect on total and LDL-cholesterol

**Figure 5:**
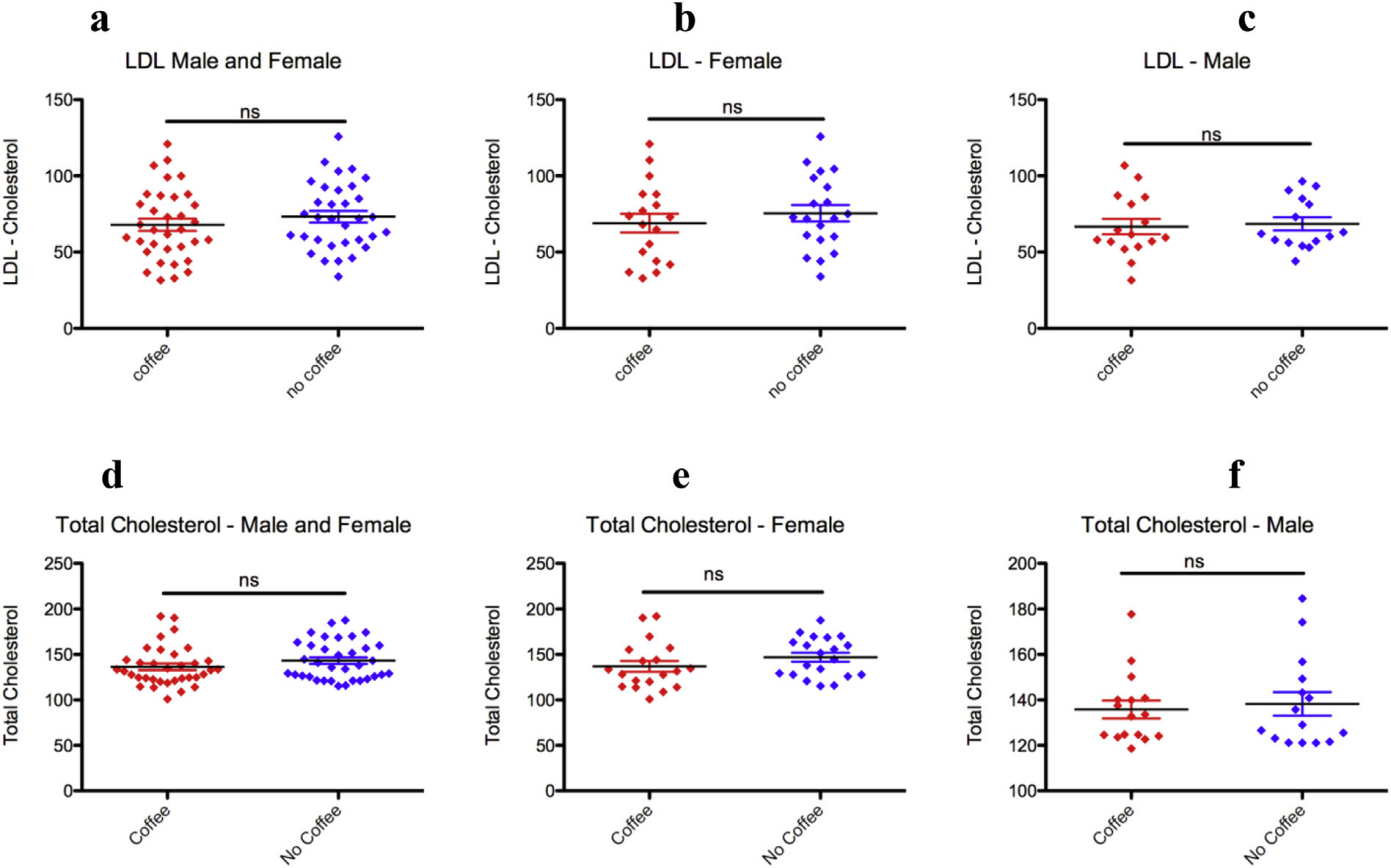
Coffee consumption has no effect on LDL-cholesterol and total cholesterol levels. (a) Coffee consumption has no effect on serum LDL-cholesterol levels both in females (b) and males (c). (d) Coffee consumption has no effect on serum total cholesterol levels both in females (e) and males (f). (mean ± SD, unpaired T-test).

To determine the effect of coffee consumption on the cholesterols positively associated with CVD, LDL-cholesterol and total cholesterol level were measured in coffee drinkers and non-drinkers. However, coffee consumption had no effect on both total cholesterol (Figure 5a-c) and LDL-cholesterol level (Figure 5d-f) both in male and female participants.

### 3.9 Coffee consumption has no effect on fasting blood glucose and insulin level

**Figure 6:**
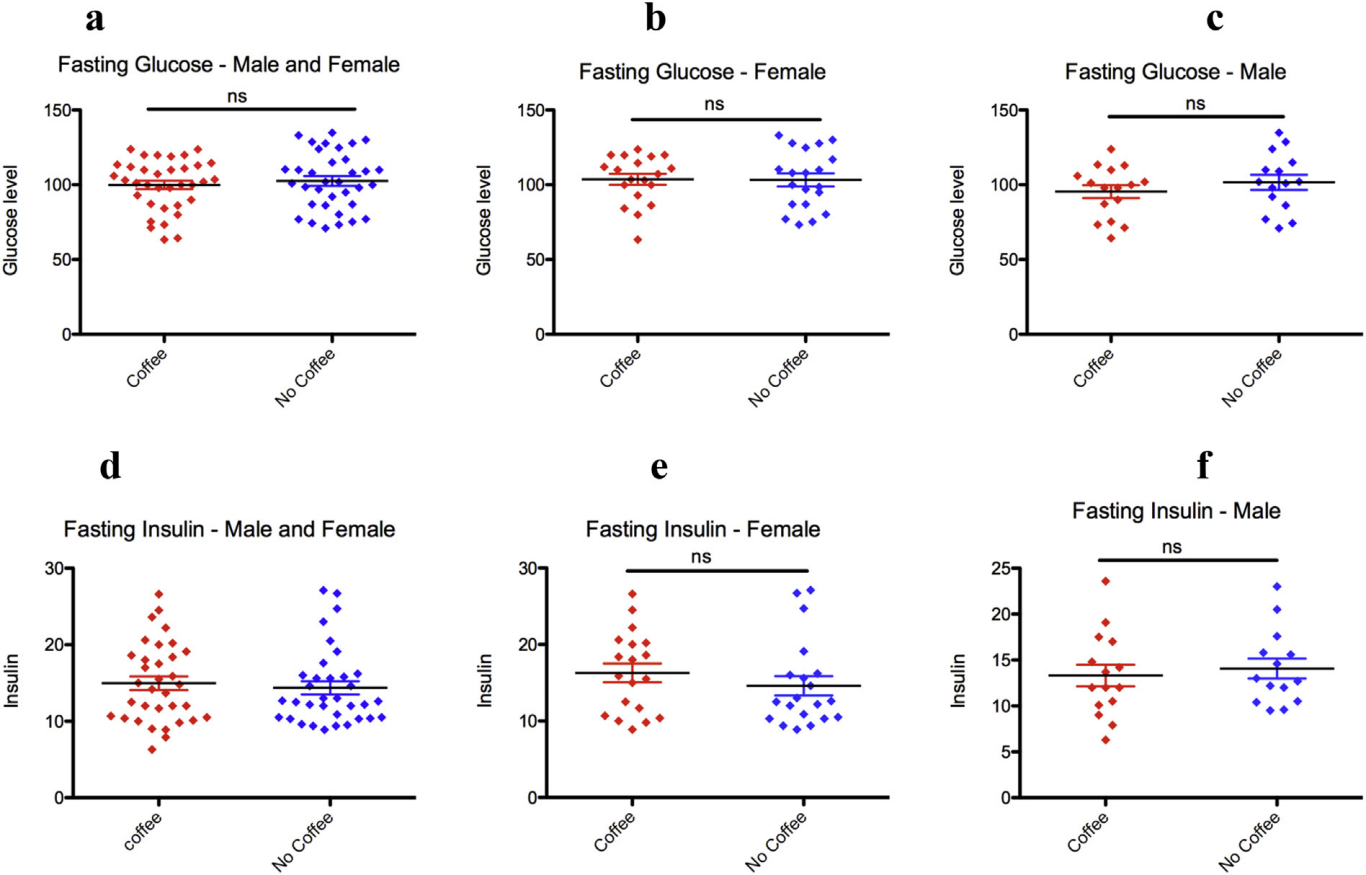
Coffee consumption has no effect on fasting glucose and insulin level. (a) coffee consumption did not affect fasting blood glucose levels both in female (b) and male (c). (d–f) as in (a) for fasting insulin level. (mean ± SD, unpaired T-test).

Coffee consumption has been reported to affect blood glucose level and associated with high insulin sensitivity (Arnlöv et al., 2004). Furthermore, coffee consumption decreases risk of type 2 diabetes (Tuomilehto et al., 2004, van Dam and Feskens, 2002). It has also been shown that coffee consumption improves insulin resistance (Lecoultre et al., 2014). In the present study coffee consumption was not associated with fasting blood glucose level and plasma insulin level. Further, Homeostatic model assessment (HOMA) insulin resistance (HOMA-IR) index is within the normal range and has no significant difference among coffee drinkers and nondrinkers (Figure 6).

## 4 Discussion

The present study conducted in Addis Ababa has shown the association between Ethiopian coffee Arabica consumption and the risk of CVDs among apparently healthy individuals. The risk of CVDs for this study was assessed by serum lipid profile, fasting and post-loading glucose and insulin with adjusted confiding factors, such as BMI, age and sex.

The main finding of this study was that consumption of Ethiopian origin arabica coffee leads to a significant increase in the serum FFA and HDL-cholesterol as well as a significant decrease in TAG but has no significant effect both in the total and LDL-cholesterol among the study population following adjustment of the results for confounding factors. Significantly increases of blood free fatty acid may be due to the lipolytic effect of caffeine through increasing prolongs action cAMP. Caffeine facilitates lipolysis through the stimulation of cortisol release and inhibition of adenosine A1 receptors on adipocytes, which results in increased FFA in the blood. None of the participants in this study consumed decaffeinated coffee. It would be interesting to compare, in the future, the serum lipid profiles of caffeinated and decaffeinated coffee drinkers to determine if the effect is due to caffeine. One recent review showed that consumption of caffeinated beverages like coffee and tea could have a beneficial effect in the risk of getting CVDs (Voskoboinik et al., 2019). Our finding is consistent with previous reports from the USA and Turkey, which showed that habitual coffee consumption has no significant effect on serum levels of TC and LDL-C but significantly decreases TAG (Richardson et al., 2009). The present study was also in line with a study done in Scottish, which revealed that a high intake of coffee had a statistically significant association with increased HDL-C and decreased TAG (Lopez-Garcia et al., 2006). A large meta-analysis report also showed that coffee consumption significantly increases TC, LDL-C and TAG with no effect in HDL (Karabudak et al., 2015). The effect of coffee on human health is reported to be dependent on the type of coffee and preparation methods. For example, filtered coffee has a lower effect size than boiled or unfiltered coffee on cholesterol level (Mostofsky et al., 2012). All the subjects in this study had exactly the same preparation method. Therefore, the HDL rising effect reported here might be associated with the type of coffee, Ethiopian Arabica coffee. However, this needs further investigation in a controlled diet and comparison of the content of the Ethiopian origin arabica coffee with other types of coffees.

The effect of long-term coffee consumption on lipid profile and its associated effect on the risks of CVD and other chronic metabolic disorders remains controversial (Malerba et al., 2013, Rebello and van Dam, 2013; Ding et al., 2014, Rodríguez-Artalejo and López-García, 2018). Depending on the type of study, study location, type of coffee and preparation methods different reports came out with different outcomes. For example, a meta-analysis of several randomized controlled trials showed that consumption of boiled coffee increased serum TC and LDL-C, while the consumption of filtered coffee resulted in a relatively smaller effect on serum TC levels (Jee et al., 2001). The mechanism for the increase in both total and LDL cholesterol following coffee consumption is due to the presence of the diterpenes cafestol and kahweol in coffee bean (Orozco-Beltran et al., 2017). The mechanism in which kahweol, a diterpene found in coffee beans, increases cholesterol levels is not well investigated. Coffee contents of chlorogenic and caffeic acid, polyphenols, caffeine and caffeine metabolites, such as di-and mono-methylxanthines, flavonoids, melanoidins and furans, pyroles and maltol have a cholesterol-lowering effect. Thus, components of coffee could have antioxidant activity and may have a protective role in the risk of CVDs (Dybkowska et al., 2017). The quantity of coffee consumed has also been demonstrated as a major important determinant of serum lipid levels. Therefore, the amount of coffee consumed and different coffee type's effects on lipid profile and risks of CVDs needs to be well investigated.

In this study, Ethiopian Arabic coffee consumption demonstrated to significantly increases serum HDL-cholesterol. In line with this, a study done in the Scottish population for 8 years suggested that higher coffee consumption was associated with increased HDL (Rodríguez-Artalejo and López-García, 2018). A meta-analysis of several large cohort studies suggested that coffee consumption decreased the risk of coronary heart disease (Liu et al., 2013). Furthermore, another large epidemiological study suggested that habitual coffee drinkers have a lower risk of cardiovascular and all-cause mortality (Liu et al., 2013). In addition, a dose-response meta-analysis stated that coffee consumption is inversely associated with all-cause and CVD mortality (Crippa et al., 2014). The presence of various biologically active molecules in coffee is believed to be a protective and detrimental factor on the risk of CVDs (Celik et al., 2010; Riksen et al., 2009; O'Keefe et al., 2018).

Plasma glucose level was determined in both coffee drinkers and non-drinkers to determine the correlation between coffee consumption and an increase in plasma FFA. However, no effects of coffee consumption were observed on the fasting blood glucose levels. Next, to determine if coffee consumption or the associated increase in plasma FFA affected insulin level independent of glucose level, plasma insulin level was measured. However, the fasting insulin level was not affected by coffee consumption. Moreover, 30 min and 2 h of glucose and insulin values, as well as the insulinogenic index derived from oral glucose tolerance tests were not affected by coffee consumption. There are several recent evidences that shows that coffee consumption increases insulin activity (Gonzalez de Mejia and Ramirez-Mares, 2014). On the other hand, coffee consumption has been associated with an increase in insulin resistance (Beaudoin and Graham, 2011). Therefore, to determine if there is any insulin resistance due to coffee consumption, HOMA was applied and found to be no significance association compared to non-coffee drinkers. In summary, Ethiopian Arabica coffee might have an advantageous effect on the risk of CVDs. However, the correlations between coffee consumption and serum lipid levels and CVDs require further investigation through epidemiological studies with a larger sample size.

## 5 Conclusion

The current study revealed that consumption of arabica coffee increases free fatty acid and HDL compared to non-drinkers. Further, coffee consumption decreases serum TAG level but no significant effect on serum LDL level and insulin sensitivity. Arabica coffee has a significant amount of effect on the lipid profile with a limited effect on glucose metabolism. The effect of coffee consumption on serum FFA was sex independent whereas the effect on TAG was sex dependent. It would be interesting to investigate the effect of sex hormones on coffee and lipid metabolism. Coffee has been shown to improve health span and delay age related diseases in model organism (Sutphin et al., 2012). lipid metabolism is an important regulator of health and lifespan (Molla et al., 2020; Admasu et al., 2018a; Admasu et al., 2018b). Therefore, it would be interesting to investigate the mechanistic link between coffee consumption and lipid metabolism and lifespan.

### 5.1 Limitation

An association observed in a cross-sectional study may not indicate a causal relationship. The study was carried out only in Addis Ababa, which may not be representative of Ethiopian residents or the general population.

## Declarations

### Author contribution statement

Gizaw M. Gebeyehu, Dereje G. Feleke: Conceived and designed the experiments; Performed the experiments; Analyzed and interpreted the data; Contributed reagents, materials, analysis tools or data; Wrote the paper.

Meseret D. Molla: Analyzed and interpreted the data; Wrote the paper.

Tesfahun D. Admasu: Conceived and designed the experiments; Analyzed and interpreted the data; Contributed reagents, materials, analysis tools or data; Wrote the paper.

### Funding statement

This work was supported by Arsi University and 10.13039/501100007941Addis Ababa University.

### Competing interest statement

The authors declare no conflict of interest.

### Additional information

No additional information is available for this paper.

## Appendix A Supplementary data

Questinary
