# Supplementary material for: Effect of habitual consumption of Ethiopian Arabica coffee on the risk of cardiovascular diseases among non-diabetic healthy adults
Source: Heliyon. 2020 Sep 15;6(9):e04886. doi: 10.1016/j.heliyon.2020.e04886 (PMC7501436; doi:10.1016/j.heliyon.2020.e04886)
Supplement: Supplementary file 1 — Questinary [file mmc1.docx]

**Appendix I Questionnaire**

1. Personal identification
   1. Full name of the subject____________________________________
   2. Subject identification number___________________
2. Demographic details

| **a)** | Date of birth________ | | | | |  |  |  |
| --- | --- | --- | --- | --- | --- | --- | --- | --- |
|  |  |  |  |  |  |  |  |  |
| **b)** | Gender | 1. Male | |  |  |  | 2. Female |  |
|  |  |  |  |  |  |  |  |  |
| **c)** | Address: | City______________ | | | |  |  |  |
|  |  | Woreda__________________ | | | | | |  |
|  |  | Kebele___________________ | | | | | |  |
|  |  | Phone number_______________ | | | | | |  |
| 3. Group: Study | |  | Control | | |  |  |  |
|  |  |  |  |  |  |  |  |  |
|  |  |  |  |  |  |  |  |  |

1. Life style
   1. Daily coffee consumption (Cups per day)

| 1. <1 | 2. 1-2 | |  | 3. 3-4 | |  |  |  | 4. ≥5 | | |  |
| --- | --- | --- | --- | --- | --- | --- | --- | --- | --- | --- | --- | --- |
|  |  |  |  |  |  |  |  |  |  |  |  |  |
| b) Daily tea consumption (cups per day) | | | | | | |  |  |  |  |  |  |
| 1. <1 |  |  |  | |  |  |  | 4. ≥5 | | | |  |
|  |  | 2. 1-2 |  | 3. 3-4 | |  |  |  |  |  |  |  |
|  |  |  |  |  |  |  |  |  | |  |  |  |
|  | | |  | |  |  |  | | |  |  |  |
| c) Current alcohol use: | | | Yes | |  |  | No | | |  |  |  |
| d) Cigarette smoking: | | | Yes | |  |  | No | | |  |  |  |
|  |  |  |  |  |  |  |  |  |  |  |  |  |
|  | | |  | |  |  |  |  | |  |  |  |
|  | | |  | |  |  |  |  | | | |  |
| f) Parental history of diabetes | | | Yes | |  |  |  | No | |  |  |  |
|  |  |  |  |  |  |  |  |  |  |  |  |  |

1. Physical
   - Weight (kg): ________
   - Height (m): ________
   - BMI (kg/m^2^): ____________
   - Waist Circumference (cm): _______
   - Hip Circumference (cm): _________
   - WHR_______
2. Vital values
   - Pulse___________________
     - Systolic/diastolic blood pressure (mmHg) _____________________
   1. Chemistry
      - Fasting plasma Glucose(mg/dl) _________________
      - Post load plasma glucose (mg/dl) ________________
      - Post load plasma insulin(µU/l)________________
      - Fasting plasma insulin(µU/l) ________________________
      - Free fatty acid(mg/dl) ________________________
      - LDL-C(mg/dl)______________________________
      - HDL-C(mg/dl)____________________________
      - Triglyceride(mg/dl)___________________
3. Any history of medical therapy
   - - 1. Current hypoglycemic therapy (oral and insulin)____________
       2. Antihypertensive or hypolipidemic__________________
       3. Other medical therapies _____________________________
4. Clinical history of major cardiovascular events

________________________________________________________________

**Appendix II**.

Information sheet and constant form for participants of the study entitled Association of coffee consumption with insulin sensitivity and plasma lipid profile among non diabetic individuals in Addis Ababa.

A- Information sheet (English Version) Addis Ababa University, Department of Biochemistry, Addis Ababa, Ethiopia

Principal Investigator: Gizaw Mamo Gebeyehu

Advisors: - Dr. Tesfahun Dessale Admasu

Name of the Sponsor: Addis Ababa University, Faculty of Medicine, Department of Biochemistry. This information sheet is prepared by researcher at AAU for a project with the aim of assessing the association of coffee consumption with insulin sensitivity and plasma lipid profile among non-diabetic individuals in Addis Ababa.

1. **Aim of the Study**

Coffee is among the most widely consumed beverage in the world including Ethiopia. Most of Ethiopian adults are consuming coffee on daily basis, because the coffee ceremony is one of the most recognizable symbols of the Ethiopian culture. So knowledge of both the positive and negative health effect of coffee is important to allow individual to make informed choice regarding to coffee consumption. A prospective study performed in the Netherlands and Japan reported that coffee consumption was association with a decrease of type – 2 – diabetes. However, a population based study of Pima Indian and Finish failed to observe a protective effect of coffee. This study will reduce the gap between two studies and provide the first data in Ethiopia whether or not coffee consumption provides protective effective against the development of the type-2-diabetes.

**2. Study design and procedure**

If you agree to take part in the study, investigators will give you written information about the study and you will be given the consent form to sign.

Investigator will ask you some questions about your general health and will take a drop of blood from your finger and assess whether you qualify to participate in the study. If you are fit for the study 5ml of blood after an over night fasting and 2 hr after you are given with 75 g oral glucose will be collected.

**3. Risk and discomfort**

Participating in this project will not cause more discomfort and no need of extra sample other than samples taken for diagnostic purpose. But, there could be minor pain and change in color of your skin following the blood drawing. The amount of blood taken from each volunteer throughout the study period is 10mL which will not affect your health. There is no major risk in participating in this

research, as the whole procedure is carried out by physician and / or health professionals following the standard good clinical practice.

**4. Benefits and Incentives**

You will have the chance to know your blood sugar level from the laboratory result. And if your result reveals any incidental health problems (Hyperglycemia) that need immediate treatment, you will be referred to an appropriate health facility. The study could benefit many peoples who are consuming coffee on daily basis. You will not be provided with any direct incentives for your participation in the research. But the cost for blood collection will be covered by the project.

**5. Confidentiality**

All information about the patients will be kept confidential. Logbooks used in the laboratory will have no names but codes. The information sheet that links the coded number to participants name will be locked inside a box and it will not be revealed to any one except the principal investigator.

**6. Right to refuse or withdraw**

You have full right to refuse or with draw from participating in this study at any time before and after consent with out explaining the reason.

**7. Whom to contact**

This study protocol is reviewed and approved by ethical committee, Addis Ababa University Medical faculty Department of Biochemistry and the Ethiopian National Ethical Research Committee (NERC). The purpose of the review by these committees is to make sure that research participants are protected from harm. For more information you can contact the chairman of the committee __________________________________________, and ________________________) Chair person the NERC.

To know more information about the study you can contact any of the following individuals:

1. IRB address (tel.No. 0115538734),mailadress ( aaumfi@ yahoo.com)
2. Ato Gizaw Mamo (mobile tel. No. 0911-80 43 67)

**Appendix IV Consent Form**

For participation as a volunteer in the research undertaking Code number_______________________________

Name of study subject________________________

I have been informed about a study that plans to correlate coffee consumption with insulin sensitivity and plasma lipids profile. For this purpose, blood needs to be taken from me. The aims of the study and the possible risks, including mild pain during blood collection were explained to me.

I am also informed that all the information contained with in the questionnaire is to be kept confidential. Moreover, I have also been well informed of my right to keep hold of information, decline to cooperate and make myself withdraw from the study.

It is, therefore, with full understanding of the situation that I gave the informed consent voluntarily to the researcher to use the blood taken from me for the investigation. Moreover, I have had the opportunity to ask questions about it and received clarification to my satisfaction. I have also been informed that the nature of the questionnaire is private.

Signature ________________ Signature __________________

(Participant) (Investigator)
